# *NlCYP4G76* and *NlCYP4G115* Modulate Susceptibility to Desiccation and Insecticide Penetration Through Affecting Cuticular Hydrocarbon Biosynthesis in *Nilaparvata lugens* (Hemiptera: Delphacidae)

**DOI:** 10.3389/fphys.2019.00913

**Published:** 2019-07-26

**Authors:** Shengyin Wang, Baoling Li, Dayu Zhang

**Affiliations:** College of Agriculture and Food Science, Zhejiang A&F University, Hangzhou, China

**Keywords:** *Nilaparvata lugens*, CYP4G, hydrocarbons, waterproofing, insecticide penetration

## Abstract

The functions of cuticular hydrocarbons (CHCs) are varied in insects, but one example is to reduce water loss. Previous work has suggested that biosynthesis of CHCs is strongly related to the CYP4G sub-family. Targeting these genes in the brown planthopper, *Nilaparvata lugens* Stål, might be a new application for integrated pest management. Therefore, we explored the functions of *CYP4G76* (GenBank: KM217045.1) and *CYP4G115* (GenBank: KM217046.1) genes in this study. The desiccation treatment (RH < 5%) for the duration of 1–3 days significantly increased the transcription level of *CYP4G76* and *CYP4G115*. RNAi through the injection of *CYP4G76* and *CYP4G115* dsRNA could significantly decrease their expression, respectively, and further reduced the biosynthesis of CHCs, i.e., saturated and straight-chain alkanes. When *CYP4G76* and *CYP4G115* were suppressed, the susceptibility of *N. lugens* nymphs to desiccation increased, due to the deficiency of the CHCs in the insect’s cuticle. When the expression of *CYP4G76* and *CYP4G115* was decreased, this resulted in an increased rate of penetration of the four insecticides: pymetrozine, imidacloprid, thiamethoxam and buprofezin. Therefore, *CYP4G76* and *CYP4G115* appear to regulate the biosynthesis of CHCs in *N. lugens* nymphs, which play a major role in protecting insects from water loss and the penetration of insecticides. *CYP4G76* and *CYP4G115* might be used as a novel target in integrated pest management to *N. lugens.*

## Introduction

Insect cuticular hydrocarbons (CHCs) are a mixture that contains many straight and branched saturated alkanes and unsaturated alkenes from C_21_ to C_37_ (Blomquist et al., 1987; Lockey, 1988). Many previous researches showed that the functions of CHCs were various (Châline et al., 2005; Blomquist and Bagneres, 2010; Balabanidou et al., 2016; Chen et al., 2016; Yu et al., 2016; Otte et al., 2018; Shahandeh et al., 2018; Wang et al., 2019), including ecology, behavior, and biochemistry. Previous researches also indicated that the CHCs are formed with long-chain fatty alcohol or aldehyde by the insect-specific CYP4G, which encodes an oxidative decarboxylase belonging to the cytochrome P450 gene family (Qiu et al., 2012; Balabanidou et al., 2016; Chen et al., 2016; Yu et al., 2016; Otte et al., 2018). Modulating CHCs biosynthesis to regulate water loss through the cuticle might be the primary role of the CYP4G subfamily (Qiu et al., 2012; Chen et al., 2016; Yu et al., 2016). Furthermore, metabolizing the hazardous materials such as insecticides is also an important function of CYP4G members (Guo et al., 2010; Martínez-Paz et al., 2012). In the subfamily of CYP4G, there are only one or very few CYP4G genes in most insect species (Feyereisen, 2011, 2012). Based on our transcriptional data and a thorough NCBI database search (Lao et al., 2015), we identified two CYP4G candidate genes: *CYP4G76* (GenBank: KM217045.1) and *CYP4G115* (GenBank: KM217046.1) in the brown planthopper, *Nilaparvata lugens* Stål, both of which may be involved in CHCs biosynthesis of *N. lugens*.

*Nilaparvata lugens* is a major pest of rice, and it causes huge economic losses worldwide both through direct feeding and as a powerful vector of plant viruses (Zhao et al., 2005). Currently, the primary management strategy against *N. lugens* is frequently used insecticides include pymetrozine, imidacloprid, thiamethoxam, and buprofezin (Smith et al., 2008; He et al., 2011; Butler et al., 2012; Liu et al., 2013). The wax layer and cement layer of the insect cuticle have a blocking effect on the invasion of insecticide, and insects with more wax are less likely to be permeated by insecticides (Sato, 1992). As CHCs are the important constituents of wax layer, inhibiting CHCs biosynthesis in *N. lugens* is likely to increase the penetration rate and to reduce the amount of insecticide used in typical management against the field-resistant population, which would be beneficial for providing an opportunity to develop new strategies with molecular tools to control *N. lugens*.

To achieve the above objectives, we explored the functions of *CYP4G76* and *CYP4G115* in *N. lugens* through RNAi technology and gas chromatography-mass spectrometry (GC-MS), and researched the effects of suppressing these two target genes on the control efficiency of insecticides.

## Materials and Methods

### *Nilaparvata lugens* Populations and Insecticides

Rice (var. ‘TN1’) plants were grown in a glass culture dish (12 cm diameter) and enclosed in nylon cages (60 cm × 60 cm × 60 cm), which were watered and fertilized as needed (Compost, COMPO Expert GmbH, Germany). An *N. lugens* was released by the College of Agriculture and Biotechnology at Zhejiang University in 2013, and was reared on the rice plants (10 cm height) at Lab of Insect Physiology, Zhejiang A&F University. Both of rice plants and *N. lugens* were maintained at the same greenhouse at 25 ± 0.5°C, RH 70 ± 5%, and with a photoperiod of 14/10 h (light/dark).

Technical grade buprofezin (98.0% pure; CAS: 69327-76-0), imidacloprid (99.9% pure; CAS: 138261-41-3), and thiamethoxam (99.0% pure; CAS: 153719-23-4) obtained from Biaozheng Chemical Company, Inc. (Xi’an, Shaanxi, China) and pymetrozine (98.6% pure; CAS: 123312-89-0) was purchased from Longdeng Chemicals Pty Ltd. (Kunshan, Jiangsu, China).

### Bioassay of Desiccation Resistance

To explore the influence of desiccation on the transcript levels of *CYP4G76* and *CYP4G115*, thirty instar *N. lugens* nymphs were exposed to the desiccation conditions. According to the method depicted by Gibbs et al. (1997), 200 g of arid allochroic silica gel (2.0–5.6 mesh, Qingdao, Shandong, China) were placed into a 2-l sealed box to decrease the relative humidity by 5% during the course of 1 h. Each biological replicate contained thirty *N. lugens* nymphs in a glass tube (30 mL) that had been sealed with nylon gauze (20 meshes). Five biological replicates and 200 g arid allochroic silica gel were put into a sealed box. The desiccation treatment lasted for 1, 2, 3 and 4 days, resulting in four allochroic silica gel treatments. As a control, *N. lugens* nymphs was placed in a glass tube and then placed in a climate chamber set at ∼70% RH. After the treatments, ten living nymphs were randomly selected from each biological replicate, the total RNA was extracted, the cDNA was synthetized, and the relative transcript level of the four allochroic silica gel treatments was compared with the control using relative quantitative PCR.

In order to explore the mortality and phenotype of *N. lugens* nymphs under a desiccation condition, the third instar nymphs were injected with 40 nL (2000 μg⋅mL^–1^) of ds*GFP*, single ds*CYP4G76*, single ds*CYP4G115*, or combined dsRNAs containing ds*CYP4G76* and ds*CYP4G115* (1:1), these injected insects were raised for 4 days to produce a new cuticle and then were kept at a desiccation condition (RH < 5%) for 1 day.

### Tissue Dissection, RNA Isolation, cDNA Synthesis, and Cloning

Before extracting the total RNA, all treated *N. lugens* nymphs were stored at −80°C in an ultra-low temperature freezer. The method and reagents for extracting the total RNA followed those outlined by Dalian Takara Co., Ltd. (Liaoning, China). The first strand of cDNA was synthesized with 500 ng total RNA and PrimeScript^*TM*^ RT reagent Kit (Takara Co., Ltd., Liaoning, China). Paired primers (10 nM) were designed and used to clone two 383 and 420 bp fragments of the target genes *CYP4G76* and *CYP4G115*, respectively. The green fluorescent protein (*GFP*, GenBank: AF372525.1) with an 864 bp fragment was used as a control. All primers were shown in Table 1.

**TABLE 1:** Primers used for PCR amplification, in RT-qPCR analysis and dsRNA synthesis.

**Application of primers**	**Primer name**	**Sequence of primers (5’-3’)**	**Products (bp)**
RT-qPCR analysis	*qCYP4G76-F*	TGTTGTTTGGCGTGGCTGTA	173
	*qCYP4G76-R*	GTCTCCCTTGTTCACGAT	
	*qCYP4G115-F*	TCGGTCCAATCCACATCTT	207
	*qCYP4G115-R*	CTCGGTCCAATCCACATC	
	*rp49-F*	CTCGTCCGCTCCTTCAATC	197
	*rp49-R*	TCGGTGACAGTGGGCGTGA	
dsRNA synthesis	ds*CYP4G76-F*	GATCACTAATACGACTCACTATAGGG TTGTTTGGCGTGGCTGTA	435
	ds*CYP4G76-R*	GATCACTAATACGACTCACTATAGGG TGGAAGGTGGGAGCAAT	
	ds*CYP4G115-F*	GATCACTAATACGACTCACTATAGGG TGGCTGAGACCCGACAT	472
	ds*CYP4G115-R*	GATCACTAATACGACTCACTATAGGG TCGGTCCAATCCACATCTT	
	ds*GFP-F*	GATCACTAATACGACTCACTATAGGG GTGGAGAGGTGAAGG	583
	ds*GFP-R*	GATCACTAATACGACTCACTATAGGG GGGCAGATTGTGTGGAC	

The PCR thermocycler parameter was as follows: (1) 94°C for 3 min; (2) 34 cycles at: 94°C for 30 s, 55°C for 30 s, 72°C for 20 s; and (3) 72°C for 5 min. All reagents were supplied by Takara Co., Ltd. (Dalian, Liaoning, China). The target fragments were retrieved from 1.0% agarose gel with Gel Extraction Kit (OMEGA Bio-tek, Norcross City, Georgia, United States), and were then cloned in pGEM-T Easy Vector (Takara Co., Ltd., Dalian, Liaoning, China), according to product manual. All positive clones for *CYP4G76* and *CYP4G115* were corroborated by DNA sequencing (Biosune Co., Ltd., Shanghai, China).

### Quantitative PCR of *CYP4G76* and *CYP4G115* mRNA

The relative expression levels of *CYP4G76* and *CYP4G115* in *N. lugens* at all life stages, and among different tissues, were measured by Bio-Rad Quantitative PCR (CFX96Touch^*TM*^ qPCR, Hercules, CA, United States). The cDNA for qPCR were obtained with the method described as PrimeScript RT reagent Kit (Takara Co., Ltd., Liaoning, China). Each qPCR date was calculated using five biological replicates, and each biological replicate contained three technical replicates. The qPCR program was as follows: (1) 94°C for 3 min, (2) 40 cycles at 94°C for 10 s, and (3) 56°C for 30 s. The ribosomal protein S3 (*rps3*; GenBank: XM_022328949) in *N. lugens* was used to normalize the transcript levels of the housekeeping gene. The *C*_*t*_ value was first normalized with *rps3* standard values and then was used to calculate the quantitative variation of target genes using the method proposed by Pfaffl (2001).

### Synthesis of dsRNA

Two fragments were selected from *CYP4G76* and *CYP4G115* as RNAi target regions, and were 383 and 420 bp, respectively (Table 1). The *GFP* gene was used as control in RNAi experiment. The promoter for the RNAi target regions and the T7 RNA polymerase promoter sequence were bound together by pGEM-T vector, and the lengths were 435 and 472 bp, respectively. The new fragment was used to amplify the target region, which was the template in dsRNA synthesis. The extra-organismal synthesis and purification of dsRNA was performed *in vitro*, and the reagent was T7 RiboMAX^*TM*^ Express RNAi Systemt (Takara Co., Ltd., Dalian, Liaoning, China). After quantifying with ultraviolet spectrophotometry at 260 nm (Lee and Schmittgen, 2006), the concentration of dsRNA was diluted to 1000 and 2000 μg⋅mL^–1^ using RNase free water (Scott et al., 2013). The dsRNA was first checked with 1% agarose gel electrophoresis and then stored at −80°C.

### Delivery of dsRNA and Detection of RNAi Efficiency

The RNAi treatment of *N. lugens* nymphs was performed by using microinjection methods. Third instar nymphs were anesthetized with CO_2_, and the prepared dsRNAs were injected into the haemolymph through the thorax ventral using a micro-injector (FemtoJet^®^ 4i, Eppendoff international trade Co., Ltd., Shanghai, China). The injected nymphs were reared on rice plants in glass bottles (8 cm diameter, 12 cm height), and were collected at each suitable time point. The method for extracting total RNA and synthesizing cDNA was the same as described. The relative transcript levels of *CYP4G76* and *CYP4G115* were measured by qPCR.

### Extraction and Quantification of CHCs

In RNAi treatment, the *GFP*, *CYP4G76*, *CYP4G115*, and commingled dsRNA containing ds*CYP4G76* and ds*CYP4G115* (1:1) were injected into third instar *N. lugens* nymphs, and hydrocarbons on newly molted *N. lugens* nymph cuticles were extracted using the procedure outlined by Young and Schal (1997).

Fifty fourth-instar nymphs and 5 mL n-hexane were put into a clear glass bottle (20 mL), and 200 ng of *n*-heneicosane was added as an internal standard. The bottle was agitated gently for 3 min to dissolve CHCs. The solution was drawn into a new chromatogram vial (20 mL) using a glass pipette. The glass bottle was rinsed twice with 3 mL *n*-hexane, and the three solutions were combined together. The combined solution (9 mL) was purified with ∼300 mg silica gel (70–230 mesh; Sigma-Aldrich, Louis, MO, United States) and poured into a clear chromatogram vial (20 mL), then taken to dryness gently with high-purity N_2_. The CHCs were re-suspended into 50 μL hexane for gas chromatography analysis.

The gas chromatograph (GC) used in this study was equipped with an ISQ single quadruple mass spectrometry (MS, Agilent 7010B; Agilent Technologies Co., Ltd., Beijing, China). The carrier gas was helium and the flow was 1 mL⋅min^–1^. We performed splitless injection of 10 μL into a 30 m × 0.32 mm × 0.25 mm capillary column (Agilent HP-5MS UI, Santa Clara, CA, United States), operated at 60°C for 2 min, then increased 5°C min^–1^ up to 320°C, where it was kept for 10 min. The injector and detector temperatures were set at 300 and 280°C, respectively. Mass detection was operated under an EI mode with a 70 eV ionization potential and a 45–650 m/z scan range at a 5 scan/s scan rate.

### Cuticular Penetration Rate of Insecticide

The *GFP*, *CYP4G76*, *CYP4G115*, and commingled dsRNA containing ds*CYP4G76* and ds*CYP4G115* (1:1) dsRNA (40 nL, 2000 μg⋅mL^–1^) were injected into the third instar *N. lugens* nymphs. The fourth instar newly molted *N. lugens* nymph were used to measure the cuticular penetrating rate of insecticides using the micro-spot method (Liu et al., 2013). Buprofezin, imidacloprid, thiamethoxam, and pymetrozine were dissolved in acetone (40 mg⋅L^–1^) and 0.5 μL of each insecticide solution was placed on the thorax cuticle using a micro-injector. After 8 h, the residue insecticide on the epidermis was eluted 3 times using 1 mL acetone. The total eluent was collected and dried with high purity N_2_. Lastly, buprofezin, imidacloprid, thiamethoxam, and pymetrozine were adusted to a volume of 100 μL using acetonitrile-water (3:7 (V/V)), methanol-dichloromethane (5:95), methanol, and n-hexane, respectively (Obana et al., 2002; Singh et al., 2004; Campbell et al., 2005; Zhang, 2007; dos Santos et al., 2008). All liquids were stored in the dark at room temperature before testing.

Imidacloprid, thiamethoxam, and pymetrozine were measured using high efficiency liquid chromatography (Segura et al., 2000). A liquid chromatographic system (Waters model 990; Milford, MA, United States) was used for the quantification and confirmation of imidacloprid, thiamethoxam, and pymetrozine, and was equipped with a Model 600E constant-flow pump, a Rheodyne six-port injection valve with a 20 ml sample loop, and a Model 990 photodiode-array detector. The spectral resolution was 1.4 nm per diode in the range 200–290 nm. HPLC separations were carried out using a Hypersyl Shandon Green Environ-C_18_-column (150 mm × 46 mm ID; 5 μm particle size). The chart speed was 0.5 cm⋅min^–1^, and the detector sensitivity was 0.02 a.u.f.s.

For pymetrozine, a carbinol-phosphate buffer (35:65 [v/v]) was used for the mobile phase, with a flow rate of 0.08 mL⋅min^–1^ (Li et al., 2011). The photometric detection was performed at 298 nm and the column temperature was 25°C. The analytical methods for imidacloprid followed a mobile phase of acetonitrile-water (20:80 [v/v]) at a flow rate of 1 mL⋅min^–1^ (Segura et al., 2000). The photometric detection was performed at 270 nm and the column temperature was 35^*o*^C. The mobile phase for thiamethoxam was carbinol-water (18:82 [v/v]), at a flow rate of 1 mL⋅min^–1^ (Rancan et al., 2006). The photometric detection was performed at 250 nm and the column temperature was 25°C.

The residual quantity of buprofezin on the cuticle of *N. lugens* nymph was measured by Shimadzu GC-17A gas chromatograph-mass spectrometry (dos Santos et al., 2008). A fused-silica column DB-5MS (30 m × 0.25 mm × 0.25 μm) (J&W Scientific, Folsom, CA, United States), was used in conjunction with helium (purity 99.999%) as carrier gas, and at a flow-rate of 1.8 mL⋅min^–1^. The column temperature was programmed as 60°C for 1 min, 270°C for 10°C⋅min^–1^, followed by 3 min of holding time at 270°C. The solvent delay was 5 min. The injector port was maintained at 250°C and 1 μL was injected during splitless mode (0.7 min). The eluent from the GC column was transferred (via a transfer line) at 280°C and fed into a 70-e Velectron-impact ionization source. Data were acquired and processed by Shimadzu class 5000 software (Shimadzu Co., Shanghai, China). The penetration rate of insecticide was calculated as follow:

(1) $$A=\frac{B-C}{B}\times 100\%$$

Where, A = penetration rate; B = the total weight of insecticide (10 ng), and C = the residue of insecticide on *N. lugens* nymph cuticle.

### Bioassay of Four Insecticides to *Nilaparvata lugens* Nymph Silenced Target Genes

The third instar *CYP4G76* and *CYP4G115* silenced *N. lugens* nymphs were obtained using microinjection methods and were used as test sample in this study. Under carbon dioxide anesthesia, a droplet (0.5 μL) of acetone insecticide solution was applied topically to the prothorax notum using a single channel adjustable range micro applicator (Eppendorf Scientific, Inc., Hamburg, Germany). Only acetone was used for the control nymphs. Each bioassay included 5 to 6 concentrations, and 23 third instar *N. lugens* nymphs were treated in each concentration. Each treatment was repeated 3 times. The treated nymphs in each concentration were reared on three rice plants (10 days) in a three plastic cups, and maintained at 27 ± 1°C at a photoperiod of 16:8 h (L:D). The mortality caused by the pymetrozine, imidaclprid, and buprofezin treatments was recorded after 4 days, and thiamethoxam after 3 days. Nymphs were considered dead if they did not move after gentle prodding with a fine brush.

### Statistical Analyses

All data are presented as the mean ± Standard Error (SE) on the basis of independent biological replicates. Statistically analyses were performed using the Statistical Package for the Social Sciences 19.0 software (SPSS Inc., Chicago, IL, United States). Significant differences between two samples and among multi-samples were determined with Student’s *t*-test and one-way ANOVA followed by the least significant difference test (LSD), respectively, and means were separated at the level *p* < 0.05. The raw data of the toxicity of four insecticides were corrected for mortality observed in the control and analyzed using the program POLO Plus 1.0 for Probit analysis.

## Results

### Spatio-Temporal Expression of *CYP4G76* and *CYP4G115*

**FIGURE 1:**
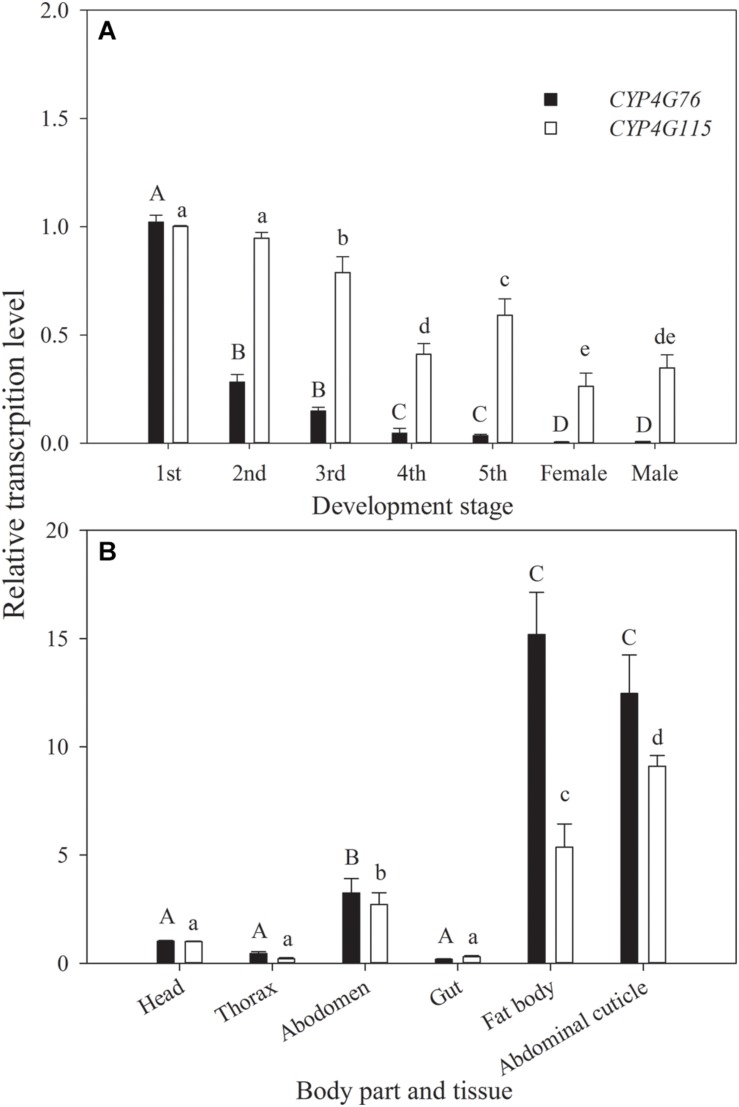
**(A)** Relative expression levels of *CYP4G76* and *CYP4G115* across developmental stages of *Nilaparvata lugens*, and **(B)** relative expression levels of *CYP4G76* and *CYP4G115* in different body parts or tissues of *N. lugens*. Mean ± SE was calculated from five biological replicates, and each biological replicate contained ten *N. lugens* nymphs or adults. Capital letters indicate significant differences between instar stages of *CYP4G76*, and lowercase letters indicate significant differences in instar stages of *CYP4G115* (ANOVA, LSD, *p <* 0.05).

The relative transcript levels of *CYP4G76* and *CYP4G115* at different developmental stages and tissues are presented in Figure 1. *CYP4G76* expression in the first instar was significantly higher than all later developmental stages (*F*_6, 28_ = 1543.723, *p* < 0.001). The relative transcript level of *CYP4G115* in most of the nymph stages was significantly higher than those in adult stages, and the transcript level in first instar larva was highest (*F*_6, 28_ = 219.890, *p* < 0.001). In terms of the effect on a specific body part, the *CYP4G76* transcript level decreased in the order of fat body, abdominal cuticle, abdomen, head, thorax and gut (*F*_5, 24_ = 178.961, *p* < 0.001). However, the expressing level of *CYP4G115* in the abdominal cuticle was significantly higher than that in the fat body, and the transcript level in other body parts and tissues decreased gradually with the same order for *CYP4G76* (*F*_5, 24_ = 423.923, *p* < 0.001).

### *CYP4G76* and *CYP4G115* Expression Under Desiccation Stress

**FIGURE 2:**
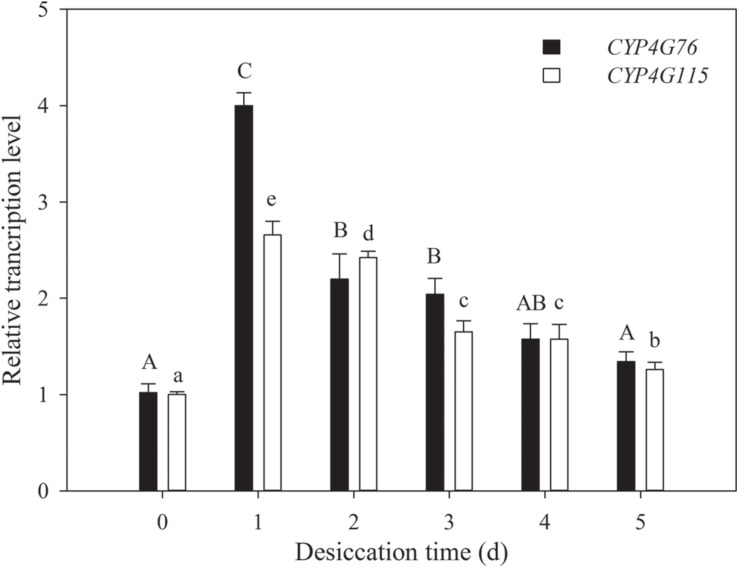
Response of *CYP4G76* and *CYP4G115* transcription levels of third instar *Nilaparvata lugens* nymph to desiccation conditions (RH < 5% for 1–5 days). The control group was at 70% RH. Mean ± SE was calculated from five biological replicates, and each biological replicate contained ten *N. lugens* nymphs. Capital letters indicate significant differences between instar stages of *CYP4G76*, and lowercase letters indicate significant differences in instar stages of *CYP4G115* (ANOVA, LSD, *p* < 0.05).

The influences of desiccation on the expression of *CYP4G76* and *CYP4G115* were investigated in third instar larvae (Figure 2). Desiccation stress for 1–3 days (RH < 5%) had a significantly higher effect on the expression of *CYP4G76* than *CYP4G115* (*F*_5, 24_ = 217.593, *p* < 0.001), though desiccation stress also significantly increased the transcript level of *CYP4G115* (*F*_5, 24_ = 187.945, *p* < 0.001).

### Silencing of *CYP4G76* and *CYP4G115* With dsRNA

**FIGURE 3:**
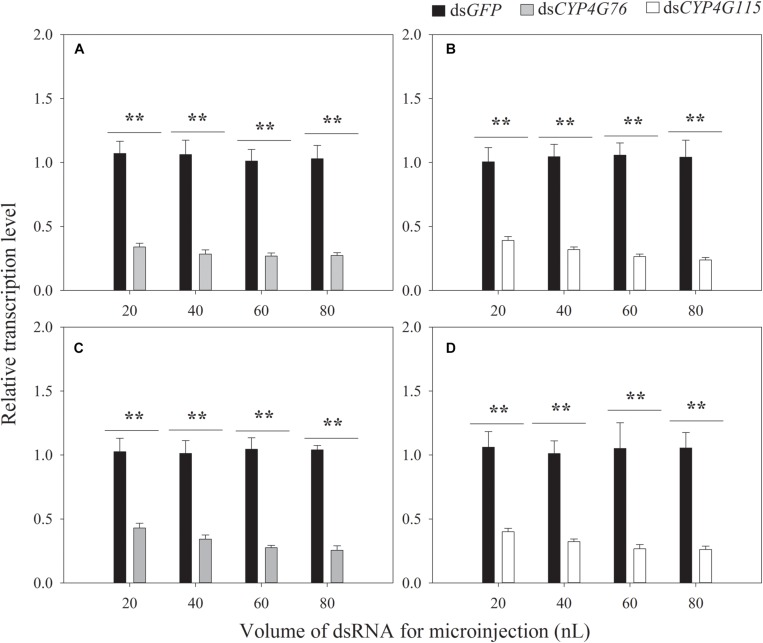
Effect of the volume of dsRNA (1000 μg⋅mL^–1^) on the expression of *CYP4G76* and *CYP4G115* in third instar *Nilaparvata lugens* nymphs 24 h following injection. **(A)** injection of *CYP4G76*, **(B)** injection of *CYP4G115* dsRNA, **(C,D)** the injection of commingled dsRNA containing ds*CYP4G76* and ds*CYP4G115* (1:1). The nymph injected dsRNA was collected at 24 h and the transcript levels of *CYP4G76* and *CYP4G115* were analyzed by qRT-PCR. Mean ± SE was calculated from five biological replicates, and each biological replicate contained ten third instar *N. lugens* nymphs. ^∗∗^ in each figure showed significant difference between treatments and control (Student’s *t*-test, *p* < 0.001).

The optimization of the volume of dsRNA is shown in Figure 3. The volumes of 20, 40, 60, and 80 nL of *CYP4G76* dsRNA (1000 μg⋅mL^–1^) significantly decreased the transcript level to 34.0% (*p* < 0.001), 28.5% (*p* < 0.001), 26.9% (*p* < 0.001) and 17.4% (*p* < 0.001), respectively. The volumes of single *CYP4G115* dsRNA in 20, 40, 60, and 80 nL (1000 μg⋅mL^–1^) were efficient for silencing of expression and resulted in a significant reduction to 39.1% (*p* < 0.001), 31.9% (*p* < 0.001), 26.5% (*p* < 0.001) and 15.9% (*p* < 0.001) in mature larva, respectively. The commingled dsRNA containing ds*CYP4G76* and ds*CYP4G115* (1:1) of 20, 40, 60, and 80 nL (1000 μg⋅mL^–1^) resulted significantly reduced the expression of *CYP4G76* to 42.9% (*p* < 0.001), 34.2% (*p* < 0.001), 27.5% (*p* < 0.001) and 17.6% (*p* < 0.001), respectively. The same commingled dsRNA silenced *CYP4G115* by 40.0% (*p* < 0.001), 32.2% (*p* < 0.001), 26.7% (*p* < 0.001), and 24.3% (*p* < 0.001), for the same 20, 40, 60 and 80 nL (1000 μg⋅mL^–1^) samples, respectively. We also observed that when the volume of dsRNA was 60 and 80 μL, it was possible for the dsRNA to overflow the injection pinhole.

**FIGURE 4:**
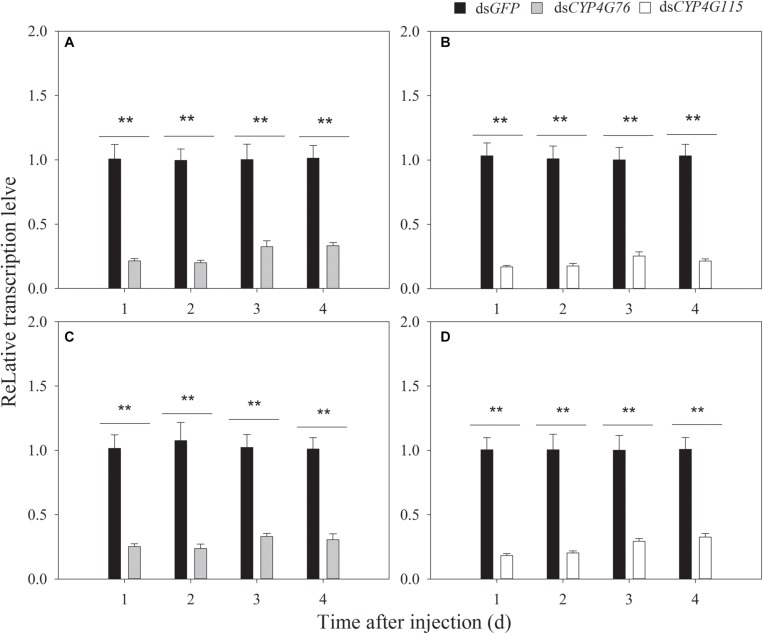
The expression of *CYP4G76* and *CYP4G115* in third and fourth instar *Nilaparvata lugens* nymphs 1–4 days after injection of single or commingled dsRNA (40 nL, 2000 μg⋅mL^–1^). **(A)** Injection of *CYP4G76* dsRNA, **(B)** Injection of *CYP4G115* dsRNA, **(C,D)** Injection of commingled dsRNA with ds*CYP4G76* and ds*CYP4G115* (1:1). The *CYP4G76* and *CYP4G115* transcript levels were analyzed by qRT-PCR. Mean ± SE was calculated from five biological replicates, and each biological replicate contained ten third instar *N. lugens* nymphs. ^∗∗^ in each figure showed significant difference between treatments and control at each time point (Student’s *t*-test, *p* < 0.001).

Due to our initial findings, we increased the concentration of dsRNA to 2000 μg⋅mL^–1^ and adjusted the injected volume to 40 nL. The RNAi efficiency of single and commingled dsRNA on the transcript expression level at 1, 2, 3, and 4 days after the microinjection is shown in Figure 4. The single injection of dsRNA reduced *CYP4G76* expression to 21.5% (*p* < 0.001), 20.1% (*p* < 0.001), 32.6% (*p* < 0.001), and 33.3% (*p* < 0.001) at 1, 2, 3, and 4 days after the injection, respectively. In addition, the similar single injection of dsRNA reduced *CYP4G115* expression to 17.8% (*p* < 0.001), 19.4% (*p* < 0.001), 26.1% (*p* < 0.001), and 21.0% (*p* < 0.001) at 1, 2, 3, and 4 days after the injection, respectively. The volume of 40 nL (2000 μg⋅mL^–1^) commingled dsRNA containing *CYP4G76* and *CYP4G115* (1:1) significantly reduced the transcript of *CYP4G76* to 20.6% (*p* < 0.001), 22.8% (*p* < 0.001), 22.4% (*p* < 0.001), and 28.9% (*p* < 0.001) at 1, 2, 3, and 4 days, respectively. The same commingled dsRNA reduced the expression of *CYP4G115* to 19.5% (*p* < 0.001), 21.4% (*p* < 0.001), 26.0% (*p* < 0.001), and 28.9% (*p* < 0.001) at 1, 2, 3, and 4 days, respectively.

### Effect of *CYP4G76* and *CYP4G115* Knockdown on CHCs Biosynthesis

**FIGURE 5:**
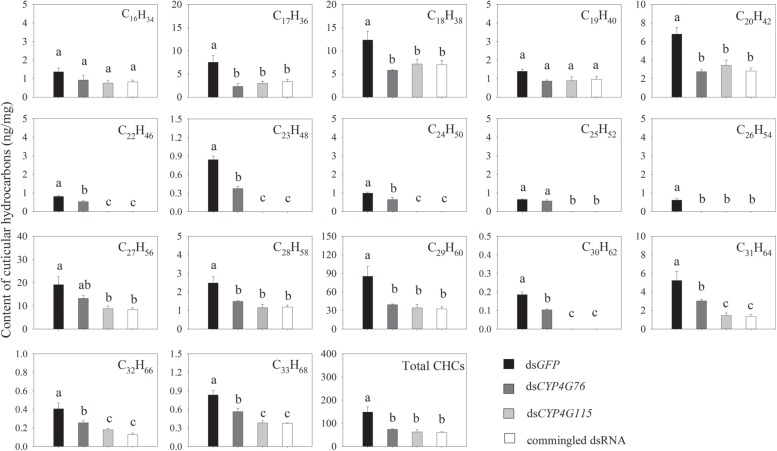
CHCs content measured from the external surface of *Nilaparvata lugens* nymphs. Commingled dsRNA contained *CYP4G76* and *CYP4G115* (1:1). Mean ± SE was calculated from three biological replicates, and each biological replicate contained fifty fourth instar *N. lugens* nymphs. Different lowercase letters in each figure showed the significant difference (ANOVA, LSD, *p* < 0.05).

The GC-MS test results indicated that the CHCs of the *N. lugens* nymph and pupa in the control treatment were a series of n-alkanes of C_16_H_34_-C_33_H_68_ (except for C_21_H_44)_ that were saturated and without methyl branched CHCs. In the control treatment, the content of these alkane groups in the CHC decreased in the following order: C_29_H_60_, C_27_H_56_, C_18_H_38_, C_31_H_64_, C_20_H_42_, C_17_H_36_, C_28_H_58_, C_16_H_34_, C_19_H_40_, C_22_H_46_, C_24_H_50_, C_24_H_50_, C_23_H_48_, C_33_H_68_, C_25_H_52_, C_32_H_66_, and C_30_H_62_ (Figure 5).

CHCs in the RNAi treatment are shown in Figure 5. The injection with single *dsCYP4G76* significantly decreased the level of the external alkanes for all alkanes: C_17_H_36_ (*F*_3, 8_ = 7.798, *p* = 0.009), C_18_H_38_ (*F*_3, 8_ = 6.026, *p* = 0.019), C_20_H_42_ (*F*_3, 8_ = 14.633, *p* = 0.001), C_22_H_46_ (*F*_3, 8_ = 93.096, *p* < 0.001), C_23_H_48_ (*F*_3, 8_ = 145.517, *p* < 0.001), C_24_H_50_ (*F*_3, 8_ = 52.087, *p* < 0.001), C_26_H_54_ (*F*_3, 8_ = 75.672, *p* < 0.001), C_28_H_58_ (*F*_3, 8_ = 9.036, *p* = 0.006), C_29_H_60_ (*F*_3, 8_ = 8.564, *p* = 0.007), C_30_H_62_ (*F*_3, 8_ = 141.144, *p* < 0.001), C_31_H_64_ (*F*_3, 8_ = 11.926, *p* = 0.003), C_32_H_66_ (*F*_3, 8_ = 11.635, *p* = 0.003) and C_33_H_68_ (*F*_3, 8_ = 18.501, *p* = 0.001). Except for C_16_H_34_ and C_19_H_40_, RNAi by silencing *CYP4G115* also significantly decreased the content of HCs, including C_17_H_36_, C_18_H_38_, C_20_H_42_, C_22_H_46_, C_23_H_48_, C_24_H_50_, C_25_H_52_ (*F*_3, 8_ = 114.969, *p* < 0.001), C_26_H_54,_ C_27_H_56_ (*F*_3, 8_ = 6.377, *p* = 0.016), C_28_H_58_, C_29_H_60_, C_30_H_62_, C_31_H_64_, C_32_H_66_, and C_33_H_68_, and C_22_H_46_, C_23_H_48_, C_24_H_50_, C_25_H_52_, C_26_H_54_, and C_30_H_62_ disappeared. It is interesting that the injection with commingled dsRNA containing ds*CYP4G76* and ds*CYP4G1115* triggered a significant reduction in all external alkanes except for C_16_H_34_ and C_19_H_40_. The amount of CHCs in three silencing treatments were significantly less than that in *GFP*-silenced control (*F*_3, 8_ = 11.399, *p* = 0.003).

### Effect of dsRNA on the Susceptibility of *N. lugens* Larva From Desiccation

**FIGURE 6:**
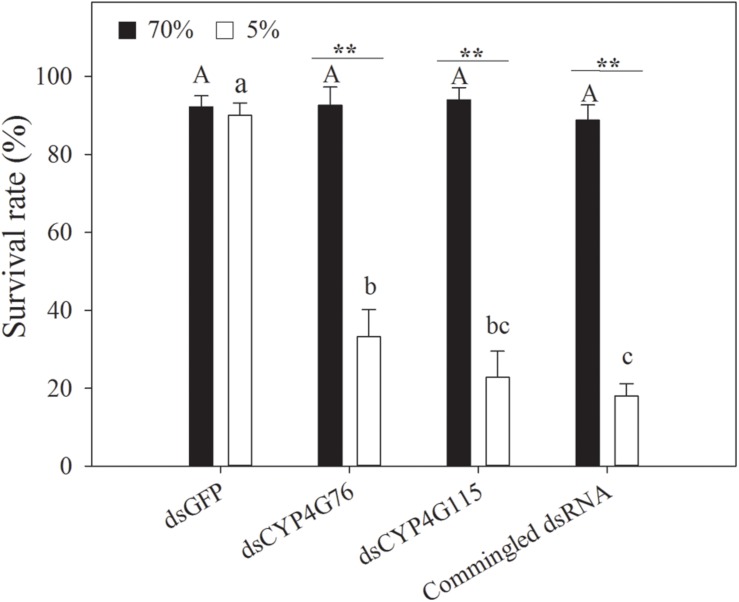
Effect of RNAi suppression of *CYP4G76* and *CYP4G115* on the survival rate of *Nilaparvata lugens* nymph following desiccation treatment (RH < 5% for 24 h). Commingled dsRNA contained *CYP4G76* and *CYP4G115* (1:1). Mean ± SE was calculated from five biological replicates, and each biological replicate contained thirty fourth instar *N. lugens nymphs*. Different capital and lowercase letters in each figure showed the significant difference (ANOVA, LSD, *p* < 0.05). ^∗∗^ in each figure showed significant difference (Student’s *t*-test, *p* < 0.001).

*CYP4G76* and *CYP4G115*-suppressed *N. lugens* larva were investigated under desiccation (RH < 5%) and control (RH = 70%) conditions (Figure 6). When the RH was at 70%, there was no significant difference in the percentage of weight loss among ds*GFP*, ds*CYP4G76*, ds*CYP4G115*, and commingled dsRNA treatments. Compared to the ds*GFP* control, the knockout of *CYP4G76* and/or *CYP4G115* significantly increased the susceptibility of the third instar to desiccation (*F*_3, 8_ = 196.594, *p* < 0.001). After injecting ds*CYP4G76*, ds*CYP4G115*, and commingled dsRNA, the survival rate of the third instar nymphs under desiccation conditions (RH < 5%) was less than those in control group (ds*CYP4G76*: *p* < 0.001; ds*CYP4G115*: *p* < 0.001; commingled dsRNA: *p* < 0.001).

**FIGURE 7:**
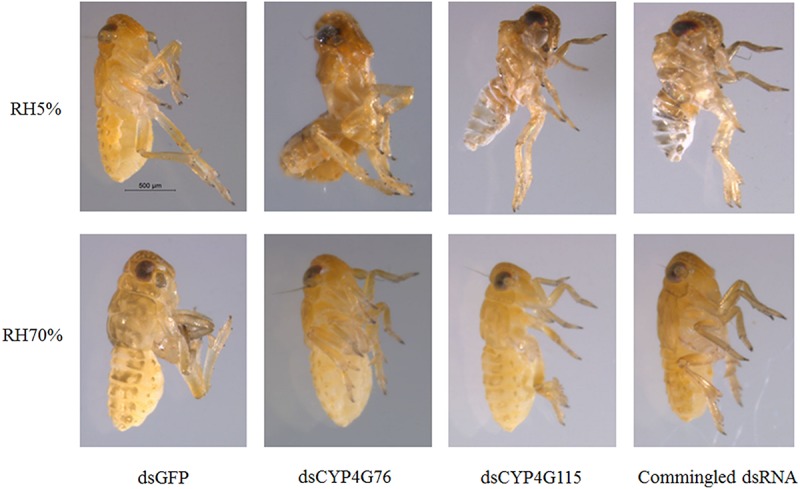
Effect of RNAi suppression of *CYP4G76* and *CYP4G115* on the phenotype of *Nilaparvata lugens* nymph following desiccation treatment (RH < 5% for 24 h). Commingled dsRNA contained *CYP4G76* and *CYP4G115* (1:1).

The nymphs that were injected with *CYP4G76* and *CYP4G115* dsRNA appeared shriveled and brittle under desiccation conditions (Figure 7). In addition, the color of the epidermis turned white and hyaline when the nymphs were injected with dsRNA containing ds*CYP4G115*. This phenomenon suggests that moisture-holding and mechanical properties of the cuticle may depend on *CYP4G76* and *CYP4G115* function.

### Effect of RNAi on the Cuticular Penetration Rate of Insecticides

**FIGURE 8:**
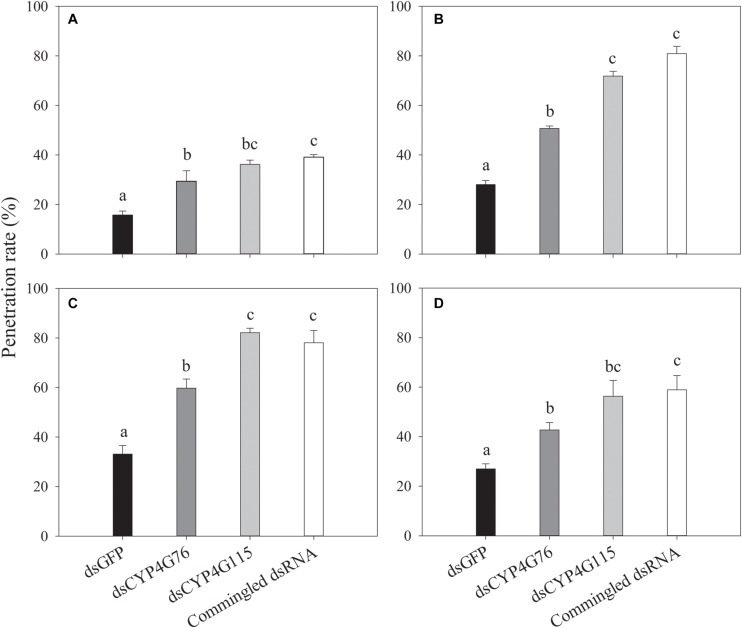
Effect of RNAi suppression of *CYP4G76* and *CYP4G115* on the penetration rate of four insecticides: **(A)** buprofezin, **(B)** imidaclprid, **(C)** thiamethoxam, and **(D)** pymetrozine, in *Nilaparvata lugens* nymphs after 8 h of exposure. Mean ± SE was calculated from three biological replicates, and each biological replicate contained ten fourth instar *N. lugens* nymphs. Different lowercase letters in each figure showed the significant difference (ANOVA, LSD, *p* < 0.05).

The penetration rates of four insecticides in the fourth instar *N. lugens* nymph are shown in Figure 8. Buprofexin has significantly greater penetration rate in *CYP4G76* and *CYP4G115*-suppressed nymphs than the *GFP* control (*F*_3, 8_ = 17.776, *p* < 0.001). The penetration rate of imidacloprid in *CYP4G76* and *CYP4G115* suppressed fourth instar nymphs was significantly greater (*F*_3, 8_ = 136.686, *p* < 0.001). Decreasing the expressing level of *CYP4G76* and *CYP4G115* significantly increased the penetrating rate of thiamethoxam in fourth instar nymphs (*F*_3, 8_ = 196.594, *p* < 0.001). The penetration rate of pymetrozine and the expressing level of the two target genes were negatively related (*F*_3, 8_ = 9.867, *p* < 0.001).

### Effect of RNAi on the Toxicity of Four Insecticides

The synergistic effects of RNAi treatment on pymetrozine, imidaclprid, thiamethoxam, and buprofezin were tested with the susceptible strains of *N. lugens* (Table 2). Results showed an increase in the synergistic effect of the treatments when *CYP4G115* was silenced, but greater synergism was found only with thiamethoxam when silencing *CYP4G76*.

**TABLE 2:** Effect of RNAi suppression of *CYP4G76* and *CYP4G115* on the synergistic ratios of four insecticides buprofezin, imidaclprid, thiamethoxam, and pymetrozine in third instar *Nilaparvata lugens* nymphs.

**Insecticide**	**Treatment**	**Slope ± SE**	**LC_50_ (95% FL)**	**Synergistic ratio**
Buprofezin	*dsGFP*	1.960 ± 0.213	1.167(0.910−−1.437)	1.000
	*dsCYP4G76*	1.835 ± 0.166	1.094(0.864−−1.368)	1.067
	*dsCYP4G115*	1.603 ± 0.155	0.431(0.348−−0.527)	2.708^*^
	Commingled dsRNA	1.931 ± 0.203	0.436(0.339−−0.547)	2.677^*^
Imidaclprid	*dsGFP*	1.474 ± 0.149	20.662(15.762−−26.509)	1.000
	*dsCYP4G76*	1.321 ± 0.143	13.290(10.452−−16.850)	1.555^*^
	*dsCYP4G115*	1.949 ± 0.206	6.531(4.945−−8.272)	3.164^*^
	Commingled dsRNA	1.602 ± 0.155	7.262(5.846−−8.881)	2.845^*^
Thiamethoxam	*dsGFP*	1.950 ± 0.203	3.141(2.527−−3.835)	1.000
	*dsCYP4G76*	1.655 ± 0.156	0.812(0.643−−1.009)	3.868^*^
	*dsCYP4G115*	1.818 ± 0.202	0.541(0.413−−0.684)	5.806^*^
	Commingled dsRNA	1.966 ± 0.171	0.299(0.249−−0.355)	10.505^*^
Pymetrozine	*dsGFP*	1.563 ± 0.132	17.725(14.951−−21.667)	1.000
	*dsCYP4G76*	1.522 ± 0.128	13.756(11.178−−16.876)	1.289
	*dsCYP4G115*	1.852 ± 0.171	5.332(4.250−−6.613)	3.324^*^
	Commingled dsRNA	1.607 ± 0.193	6.124(4.837−−7.589)	2.894^*^

FL, fiducial limits. ^*^ in each figure showed the significant difference (ANOVA, LSD, *p* < 0.05).

## Discussion

In this present study, we have successfully repressed two cytochrome P450 genes, *CYP4G76* and *CYP4G115*, using RNAi technique to determine the lethal phenotype of *N. lugens* nymphs under desiccation conditions. Functional studies have revealed that, *CYP4G76* and *CYP4G115* are critically related to CHCs biosynthesis. Reducing the *CYP4G76* and *CYP4G115* expression level through RNAi technology resulted in a higher mortality rate of *N. lugens* nymphs under desiccation conditions, and a greater penetration rate of insecticides.

We have represented that the CHCs profile of *N. lugens* is unique. Except for C_21_H_44_ and C_23_H_48_, we identified thirteen compounds of saturated C_16_H_34_-C_31_H_64_ straight chain n-alkanes and determined there was no methyl branched HC on the cuticular surface. We also showed that just five compounds (C_17_H_36_, C_18_H_38_, C_20_H_42_, C_27_H_56_ and C_29_H_60_) comprise more than 90% of the straight chain n-alkanes on the CHCs. The influence of desiccation on the mortality rate of *CYP4G76-* and *CYP4G115*-silenced *N. lugens* provided solid evidence that saturated and straight-chain CHCs are responsible for reducing the water loss from *N. lugens* cuticle by evaporation.

We found that *CYP4G76* and *CYP4G115* were highly expressed in the fat body and abdominal cuticle, which suggests that oenocytes might be precisely located in the fat body and the abdominal cuticle. Our results are different from previous studies on the fruit fly (*Drosophila melanogaster*), however, wherein *CYP4G15* was highly expressed in the larval brain and central nervous system (Maïbeche-Coisne et al., 2000; Chung et al., 2014). Therefore, future research should investigate the precise location of oenocytes by CYP4G members in *N. lugens*.

Together with previous studies (Blomquist and Bagneres, 2010; Qiu et al., 2012; Chen et al., 2016; Yu et al., 2016; Shahandeh et al., 2018), we suggested that CYP4G might have multiple functions in many insect species. Although we have found that *CYP4G76* and *CYP4G115* are related to CHCs biosynthesis, the specific enzymatic activity and precursor to CHCs are still unclear. To illuminate the functions of *CYP4G76* and *CYP4G115* and other genes belonging to the CYP4G subfamily in the Insecta class, more extensive and intensive research needs to be conducted.

Basically, the mechanisms of insect resistance consist of reducing the penetration rate of insecticide, enhancing the activity of detoxification, and target mutation (Oppenoorth, 1985; Balabanidou et al., 2016; Garrood et al., 2016, 2017). Whether insecticides penetrate the thorax cuticle into the insect body depends on the structure of the insect cuticle and the physical and chemical characteristics of the insecticides (Yang et al., 2011). Since four tested insecticides are lipophilic, the penetration rate of insecticide was mainly affected by the structure of cuticular layer. The blocking effect of insect cuticle decreased the invasion of insecticide and insects with more wax are less likely to be permeated (Sato, 1992; Balabanidou et al., 2016). In this research, we found that silencing two target genes (*CYP4G75* and *CYP4G115*) could significantly reduce the content of CHCs, further significantly increase the penetration ratio of four tested insecticides, which suggests that, reducing CHCs biosynthesis is beneficial for increasing the penetration ratio of insecticides. Furthermore, we found that suppressing *CYP4G75* and *CYP4G115* increased the synergistic ratio, and the synergistic effect of thiamethoxam was greatest, which suggested that suppressing the biosynthesis of CHCs could increase the control efficacy of insecticide on *N. lugens*. Thus, when the RNAi technique and traditional pest management strategies are combined, the field control efficiency of thiamethoxam may be the best.

Our findings confirm that *CYP4G76* and *CYP4G115* are involved in desiccation resistance of *N. lugens* by modulating CHCs production. Based on the higher mortality rate in *CYP4G76-* and *CYP4G115*-silenced *N. lugens* nymphs following the desiccation treatment, we propose that, depletion of CHCs may enhance the permeability of the cuticle, causing increased water loss, further resulting in death at last. In addition, silencing *CYP4G76* and *CYP4G115* in *N. lugens* nymphs increased penetration rates and synergistic effect of thiamethoxam was best, which suggested that it would be interesting to investigate the field control efficiency of thiamethoxam. Furthermore, *CYP4G76* and *CYP4G115* might be a promising RNAi candidate for providing an environmentally responsible approach to managing *N. lugens* populations. Since it was only found in insects (Qiu et al., 2012), targeting the insect-specific CYP4G gene might develop into a completely new application for integrated pest management.

## Author Contributions

SW and DZ designed the experiments and wrote the manuscript. SW and BL conducted the experiments. SW, BL, and DZ conducted the data analysis.

## Conflict of Interest Statement

The authors declare that the research was conducted in the absence of any commercial or financial relationships that could be construed as a potential conflict of interest.

## References

[B1] Balabanidou V. Kampouraki A. MacLean M. Blomquist G. J. Tittiger C. Juárez M. P. (2016). Cytochrome P450 associated with insecticide resistance catalyzes cuticular hydrocarbon production in Anopheles gambiae. *Proc. Natl. Acad. Sci. U.S.A.* 113 9268–9273. 10.1073/pnas.1608295113 27439866 PMC4995928

[B2] Blomquist G. J. Bagneres A. G. (2010). *Insect Hydrocarbons: Biology, Biochemistry, and Chemical Ecology.* New York, NY: Cambridge University Press, 10.1007/978-3-319-40740-1_7

[B3] Blomquist G. J. Nelson D. R. Renobales M. D. (1987). Chemistry, biochemistry, and physiology of insect cuticular lipids. *Arch. Insect Bioche.* 6 227–265. 10.1002/arch.940060404

[B4] Butler C. D. Walker G. P. Trumble J. T. (2012). Feeding disruption of potato psyllid, Bactericera cockerelli, by imidacloprid as measured by electrical penetration graphs. *Entomol. Exp. Appl.* 142 247–257. 10.1111/j.1570-7458.2012.01219.x

[B5] Campbell S. Chen L. Yu J. Li Q. X. (2005). Adsorption and analysis of the insecticides thiamethoxam and indoxacarb in hawaiian soils. *J. Agr. Food Chem.* 53 5373–5376. 10.1021/ac00199a724 15969521

[B6] Châline N. Sandoz J. C. Martin S. J. Ratnieks F. L. Jones G. R. (2005). Learning and discrimination of individual cuticular hydrocarbons by honeybees (Apis mellifera). *Chem. Senses* 30 327–335. 10.1093/chemse/bji027 15788713

[B7] Chen N. Fan Y. L. Bai Y. Li X. D. Zhang Z. F. Liu T. X. (2016). Cytochrome p450 gene, cyp4g51, modulates hydrocarbon production in the pea aphid, *Acyrthosiphon pisum*. *Insect Biochem. Molec.* 76 84–94. 10.1016/j.ibmb.2016.07.006 27425674

[B8] Chung H. Loehlin D. W. Dufour H. D. Vaccarro K. Millar J. G. Carroll S. B. (2014). A single gene affects both ecological divergence and mate choice in *Drosophila*. *Science* 343 1148–1151. 10.1126/science.1249998 24526311

[B9] dos Santos T. F. Aquino A. Dórea H. S. Navickiene S. (2008). MSPD procedure for determining buprofezin, tetradifon, vinclozolin, and bifenthrin residues in propolis by gas chromatography-mass spectrometry. *Anal. Bioanal. Chem.* 390 1425–1430. 10.1007/s00216-007-1813-y 18193409

[B10] Feyereisen R. (2011). Arthropod CYPomes illustrate the tempo and mode in P450 evolution. *BBA-Proteins Proteom* 1814 19–28. 10.1016/j.bbapap.2010.06.012 20601227

[B11] Feyereisen R. (2012). “Insect CYP genes and P450 enzymes,” in *Insect Molecular Biology and Biochemistry*, ed. Gilbert L. I. (Amsterdam: Elsevier BV), 236–316. 10.1016/B978-0-12-384747-8.10008-X

[B12] Garrood W. T. Zimmer C. T. Gorman K. J. Nauen R. Bass C. Davies T. G. E. (2016). Field-evolved resistance to imidacloprid and ethiprole in populations of brown planthopper Nilaparvata lugens collected from across South and East Asia. *Pest Manag. Sci.* 72 140–149. 10.1002/ps.3980 25612154 PMC4964955

[B13] Garrood W. T. Zimmer C. T. Gutbrod O. Lüke B. Williamson M. S. Bass C. (2017). Influence of the RDL A301S mutation in the brown planthopper Nilaparvata lugenson the activity of phenylpyrazole insecticides. *Pestic. Biochem. Phys.* 142 1–8. 10.1016/j.pestbp.2017.01.007 29107231 PMC5672059

[B14] Gibbs A. G. Chippindale A. K. Rose M. R. (1997). Physiological mechanisms of evolved desiccation resistance in Drosophila melanogaster. *J. Exp. Biol.* 200 1821–1832. 10.1142/9789812567222_0010 9225453

[B15] Guo G. Geng Y. Huang D. Xue C. Zhang R. (2010). Level of CYP4G19 expression is associated with pyrethroid resistance in *Blattella germanica*. *J. Parasitol Res* 2010 517–534. 10.1155/2010/517534 20700426 PMC2911607

[B16] He Y. Chen L. Chen J. Zhang J. F. Chen L. Z. Shen J. L. (2011). Electrical penetration graph evidence that pymetrozine toxicity to the rice brown planthopper is by inhibition of phloem feeding. *Pest Manag. Sci.* 67 483–491. 10.1002/ps.2098 21254329

[B17] Lao S. H. Huang X. H. Huang H. J. Liu C. W. Zhang C. X. Bao Y. Y. (2015). Genomic and transcriptomic insights into the cytochrome P450 monooxygenase gene repertoire in the rice pest brown planthopper, *Nilaparvata lugens*. *Genomics* 106 301–309. 10.1016/j.ygeno.2015.07.010 26234643

[B18] Lee E. J. Schmittgen T. D. (2006). Comparison of RNA assay methods used to normalize cDNA for quantitative real-time PCR. *Anal. Biochem.* 357 299–301. 10.1016/j.ab.2006.06.011 16930526

[B19] Li C. Yang T. Huangfu W. Wu Y. (2011). Residues and dynamics of pymetrozine in rice field ecosystem. *Chemosphere* 82 901–904. 10.1016/j.chemosphere.2010.10.053 21074245

[B20] Liu J. Wu S. S. Meng L. Li B. P. (2013). Effects of CO2 concentration and pesticide resistance on penetration behaviors in *Nilaparvata lugens* (Homoptera:Delphacidae). *Sci. Agric. Sin.* 46 4471–4477. 10.3864/j.issn.0578-1752.2013.21.009

[B21] Lockey K. H. (1988). Lipids of the insect cuticle: origin, composition and function. *Comp. Biochem. Phys. B* 89 595–645. 10.1016/0305-0491(88)90305-7 18413656

[B22] Maïbeche-Coisne M. Monti-Dedieu L. Aragon S. Dauphin-Villemant C. (2000). A new cytochrome P450 from *Drosophila melanogaster*, CYP4G15, expressed in the nervous system. *Biochem. Bioph. Res. Co.* 273 1132–1137. 10.1006/bbrc.2000.3058 10891384

[B23] Martínez-Paz P. Morales M. Martínez-Guitarte J. L. Morcillo G. (2012). Characterization of a cytochrome P450 gene (CYP4G) and modulation under different exposures to xenobiotics (tributyltin, nonylphenol, bisphenol A) in Chironomus riparius aquatic larvae. *Comp. Biochem. Phys. C* 155 333–343. 10.1016/j.cbpc.2011.10.001 22019333

[B24] Obana H. Okihashi M. Akutsu K. Kitagawa Y. Hori S. (2002). Determination of acetamiprid, imidacloprid and nitenpyram residues in vegetables and fruits by high-performance liquid chromatography with diode-array detection. *J. Agric. Food Chem.* 50 4464–4467. 10.1021/jf025539q 12137461

[B25] Oppenoorth F. J. (1985). “Biochemistry and genetics of insecticide resistance,” in *Comprehensive Insect Physiology, Biochemistry and Pharmacology*, eds Kerhut G. A. Gileert L. I. (Oxford: Pergamon Oxford), 731–773.

[B26] Otte T. Hilker M. Geiselhardt S. (2018). Phenotypic plasticity of cuticular hydrocarbon profiles in insects. *J. Chem. Ecol.* 44 235–247. 10.1007/s10886-018-0934-4 29468480

[B27] Pfaffl M. W. (2001). A new mathematical model for relative quantification in realtime RT-PCR. *Nucleic Acids Res.* 29:e45. 10.1093/nar/29.9.e45 11328886 PMC55695

[B28] Qiu Y. Tittiger C. Wicker-Thomas C. Le Goff G. Young S. Wajnberg E. (2012). An insect-specific P450 oxidative decarbonylase for cuticular hydrocarbon biosynthesis. *Proc. Natl. Acad. Sci. U.S.A.* 109 14858–14863. 10.1073/pnas.1208650109 22927409 PMC3443174

[B29] Rancan M. Sabatini A. G. Achilli G. Galletti G. C. (2006). Determination of Imidacloprid and metabolites by liquid chromatography with an electrochemical detector and post column photochemical reactor. *Anal. Chim. Acta* 555, 20–24. 10.1016/j.aca.2005.08.058 16735040

[B30] Sato K. (1992). Partitioning of insecticide between insect surface wax and water as a factor of toxicity. *J. Soc. Appl. Entomol. Zool.* 27 287–289. 10.1303/aez.27.287

[B31] Scott J. G. Michel K. Bartholomay L. C. Siegfried B. D. Hunter W. B. Smagghe G. (2013). Towards the elements of successful insect RNAi. *J. Insect Physiol.* 59 1212–1221. 10.1016/j.jinsphys.2013.08.014 24041495 PMC3870143

[B32] Segura C. A. Cruces-Blanco C. Pérez D. S. Fernández G. A. (2000). Determination of imidacloprid and its metabolite 6-chloronicotinic acid in greenhouse air by high-performance liquid chromatography with diode-array detection. *J. Chromatogr. A.* 869 497–504. 10.1016/S0021-9673(99)00879-1 10720263

[B33] Shahandeh M. P. Pischedda A. Turner T. L. (2018). Male mate choice via cuticular hydrocarbon pheromones drives reproductive isolation between *Drosophila species*. *Evolution* 72 123–135. 10.1111/evo.13389 29098691 PMC5760347

[B34] Singh S. B. Foster G. D. Khan S. U. (2004). Microwave-assisted extraction for the simultaneous determination of thiamethoxam, imidacloprid and carbendazim residues in fresh and cooked vegetable samples. *J. Agric. Food Chem.* 52 105–109. 10.1021/jf030358p 14709021

[B35] Smith J. A. Pereira R. M. Koehler P. G. (2008). Relative repellency and lethality of the neonicotinoids thiamethoxam and acetamiprid and an acetamiprid/bifenthrin combination to Reticulitermes flavipes termites. *J. Econ. Entomol.* 101 1881–1888. 10.1603/0022-0493-101.6.1881 19133470

[B36] Wang S. Y. Price J. H. Zhang D. (2019). Hydrocarbons catalysed by *TmCYP4G122* and *TmCYP4G123* in *Tenebrio molitor* modulate the olfactory response of the parasitoid *Scleroderma guani*. *Insect Mol. Biol.* 10.1111/imb.12581 [Epub ahead of print]. 30843299

[B37] Yang J. Wang Z. Yao A. Q. (2011). Insects resistance bioassay and insecticide penetration biology. *Chin. J. Appl. Entomol.* 48 421–425. 10.1017/S0022112010006427

[B38] Young H. P. Schal C. (1997). Cuticular hydrocarbon synthesis in relation to feeding and developmental stage in nymphs of *Blattella germanica* (Dictyoptera: Blattellidae). *Ann. Entomol. Soc. Am.* 90 655–663. 10.1093/aesa/90.5.655

[B39] Yu Z. Zhang X. Wang Y. Moussian B. Zhu K. Y. Li S. (2016). LmCYP4G102: an oenocyte-specific cytochrome P450 gene required for cuticular waterproofing in the migratory locust, *Locusta migratoria*. *Sci. Rep.* 6:29980. 10.1038/srep29980 27444410 PMC4957221

[B40] Zhang X. (2007). Efficient high-performance liquid chromatography with liquid-liquid partition cleanup method for the determination of pymetrozine in tobacco. *Ann. Chim.* 97 295–301. 10.1002/adic.200790015 17696008

[B41] Zhao Y. Huang F. K. Dong X. L. Pang X. F. (2005). HPLC analysis of rice variety resistance to different biotypes of Nilaparvata lugens. *J. South China Agr. Univ.* 26 52–55. 10.1360/982004-755

